# Factors Contributing to the Recurrence of Chronic Rhinosinusitis With Nasal Polyps After Endoscopic Sinus Surgery: A Systematic Review

**DOI:** 10.7759/cureus.67910

**Published:** 2024-08-27

**Authors:** Sarah H Abuduruk, Bayan K Sabb Gul, Shuruq M AlMasoudi, Enas H Alfattani, Mouaz A Mohammad, Hind M Alshehri, Ashwaq D Alosaimi, Rakan F Almnjwami, Johara A Alnafie, Ali N Jabbari, Abdulaziz H Althumali

**Affiliations:** 1 Department of Otolaryngology, Alhada Armed Forces Hospital, Taif, SAU; 2 Department of Otolaryngology, Al-Noor Specialized Hospital, Makkah, SAU; 3 Department of Otolaryngology, College of Medicine, Majmaah University, Al-Majmaah, SAU; 4 Department of Otolaryngology, Alhada Armed Forces Hospital, Makkah, SAU; 5 Department of Otolaryngology, King Abdullah Medical City, Makkah, SAU

**Keywords:** edema, relapse, recurrence, nasal polyps, endoscopic sinus surgery, chronic rhinosinusitis

## Abstract

Chronic rhinosinusitis with nasal polyposis (CRSwNP) occurs due to the inflammation of sinonasal tissue. Cases of CRSwNP more commonly demand revision endoscopic sinus surgery (ESS) as compared to patients without polyposis. The recurrence rate varies widely depending on various factors, such as the extent of surgery, patient compliance with postoperative care, and the severity of the underlying disease. Studies conducted on chronic rhinosinusitis (CRS) patients showing recurrence or relapse of nasal polyps post endoscopic sinus surgery were included. We used the modified Newcastle-Ottawa scale (NOS) for cross-sectional studies and cohort studies. Only 15 articles met our inclusion and exclusion criteria after the full-text screening. The studies enrolled participants between 2009 and 2022, including 2,515 ESS patients. The mean age of the included subjects ranged between 37.1 and 57.57 years. In conclusion, CRSwNP is a chronic inflammatory disease that can impose a significant burden on patients, healthcare systems, and society. Asthma, aspirin intolerance, peripheral eosinophilia, interleukin-5 (IL-5) expression, T2 profile, and intense sinus opacification have been noted to be independent predictors of the condition in different studies. Recurrent polyposis in CRS signals a more aggressive disease course, requiring close follow-up and revision surgeries in the long run.

## Introduction and background

Chronic rhinosinusitis (CRS) is defined as an inflammation of the nose and paranasal sinuses. With or without nasal polyps, it has been defined in the European Position Paper on Rhinosinusitis and Nasal Polyps (EPOS) 2020 as the presence of two or more symptoms: a) either nasal blockage or obstruction or congestion or nasal discharge (anterior/posterior nasal drip), b) with or without facial pain/pressure, c) with or without a reduction or loss of smell. These symptoms should be present for 12 weeks or longer and validated by telephone or face-to-face interview [1]. Several epidemiological studies have stated that the prevalence of CRS ranges globally between 4.6% and 12% [2, 3]. This condition has two different modes of presentation and is commonly categorized as CRS with nasal polyps (CRSwNP) or CRS without nasal polyps (CRSsNP). In CRSwNP, the most severe and prevalent symptoms are nasal obstruction and changes in the sense of smell and taste. Although CRSsNP patients also experience severe nasal obstruction, they report facial pain and nasal discharge to be as severe as changes in smell and taste. In general, CRS patients in secondary care awaiting surgery report mean symptom severity scores in the moderate to severe range by using a scoring system called the Sino-nasal Outcome Test (SNOT-22). This system includes a questionnaire that covers a broad range of health-related quality of life questions, with few questions that ask about nasal blockage and sense of taste or smell. The answers are converted into a score of 0-110, with a higher score indicating a greater impact on quality of life. Patients with CRS score an average SNOT-22 score of 42.0, compared to a mean score of 9.3 in the control group. Patients with CRSwNP have been found to report lower preoperative baseline scores (41.0) compared to those with CRSsNP (44.2) [4]. The severity rating of symptoms widely varies depending on the population being studied.

Nasal polyps occur due to the inflammation of the sinonasal tissue and are found in conditions such as cystic fibrosis and carcinomas. In CRSwNP, the polyposis is characterized by benign and usually bilateral growths. It has been observed that CRSwNP comprises a quarter to a third of all cases of CRS [5]. Chronic rhinosinusitis with nasal polyps commonly occurs in older adults, with the mean age of onset being 42 years, and is typically diagnosed between the fourth and sixth decades of life. Chronic rhinosinusitis with nasal polyps shows variability in prevalence across different populations and age groups. Early Scandinavian studies revealed a prevalence of nasal polyps at 2.7% in adults, with higher rates in men, the elderly, and individuals with asthma. One of these studies involving 1,387 adults found that polyps were more common in men and those aged over 60 years [6, 7]. Similarly, Danish data estimated an incidence of 0.86 and 0.39 per 1,000 per year for males and females, respectively, leading to a prevalence of 1.92% for males and 0.78% for females over an estimated 20-year disease duration. A validated questionnaire-based study further estimated the prevalence at 2.11%, with CRSwNP more common in older adults [7]. Chronic rhinosinusitis with nasal polyps is often associated with higher BMI, smoking, and adult-onset asthma. There is significant variability in prevalence among ethnic groups, with lower rates in Asian and Hispanic populations compared to Black and Caucasian populations. Additionally, Brescia et al. found that nasal polyps recurred less frequently in elderly patients after endoscopic sinus surgery (ESS), potentially due to lower eosinophilic infiltration, which is associated with recurrence risk; they also noted that younger patients had a significantly higher proportion of allergies, although asthma prevalence was similar across age groups. Similarly, Cho et al. suggested that CRS in the elderly has a different pathogenesis, being less associated with allergies and eosinophilic infiltration but more with nasal polyp formation [8].

Chronic rhinosinusitis with nasal polyps can significantly impact a person's quality of life. The symptoms of CRSwNP can lead to physical discomfort, social isolation, and psychological distress. In addition to clinical assessment, radiologic examination is important in the diagnosis of CRSwNP as it provides objective evidence of inflamed sinonasal tissue and polyposis. This is achieved using a sinus computed tomography (CT) scan as well as nasal endoscopy [5]. It has been established using these diagnostic aids that CRSwNP patients show a more substantial presence on CT scans and poorer endoscopic scores, which translate to worse post-treatment outcomes as compared to CRSsNP, even following similar surgical intervention. Chronic rhinosinusitis with nasal polyposis is a complex disease with a high rate of recurrence, even after successful ESS. Cases of CRSwNP, therefore, more commonly demand revision ESS as compared to patients with polyposis. The recurrence rate varies widely depending on various factors, such as the extent of surgery, patient compliance with postoperative care, and the severity of the underlying disease. Several factors have been identified as risk factors for the recurrence of CRSwNP after ESS, including younger age, male gender, asthma, aspirin sensitivity, and smoking. Additionally, failure to complete the removal of polyps and underlying inflammatory disorders has also been associated with higher rates of recurrence. To reduce the risk of recurrence, postoperative medical therapy with intranasal corticosteroids, saline irrigation, and antibiotics has been recommended.

Overall, CRS results in an additional direct healthcare expenditure of €2,500 per patient annually [9]. The highest direct costs were incurred by patients who experienced recurrent polyposis after surgery. Lourijsen et al. discovered that the annual direct costs for a group of patients with CRSwNP amounted to €1,501 per patient per year [10]. Close follow-up and regular endoscopic evaluation may also be necessary to detect early signs of recurrence and prevent the progression of the disease.

## Review

Methods

Definition of Outcomes and Inclusion Criteria

Studies conducted on chronic rhinosinusitis patients showing recurrence or relapse of nasal polyps post ESS were included. The studies assessed recurrence and procedure- and patient-specific risk factors. The following clinical outcomes were reported by the studies: recurrence rate, risk factors or predictors, and follow-up period.

Search Strategy

The residency program in Saudi Arabia is collaborative and considered a regional program; therefore, there were times when authors were posted in different hospitals depending on their rotational schedule. Therefore, we conducted a comprehensive search of online databases, including PubMed, Google Scholar, the Excerpta Medica database (EMBASE), and the Cochrane Library, to identify relevant articles that met our pre-determined inclusion and exclusion criteria. The primary search terms used were 'chronic rhinosinusitis', 'endoscopic sinus surgery', and 'nasal polyps'. To ensure a thorough search, we also included the terms 'recurrence', 'relapse', and 'edema' individually.

In PubMed, the search string was ("Chronic Rhinosinusitis"[Mesh] OR "chronic rhinosinusitis") AND ("Endoscopic Sinus Surgery"[Mesh] OR "endoscopic sinus surgery") AND ("Nasal Polyps"[Mesh] OR "nasal polyps") AND ("Recurrence"[Mesh] OR "recurrence" OR "relapse" OR "edema"). In Google Scholar, we used the search string Allintitle: "chronic rhinosinusitis" AND "endoscopic sinus surgery" AND "nasal polyps" AND ("recurrence" OR "relapse" OR "edema"). For EMBASE, the search string was ('chronic rhinosinusitis'/exp OR 'chronic rhinosinusitis') AND ('endoscopic sinus surgery'/exp OR 'endoscopic sinus surgery') AND ('nasal polyps'/exp OR 'nasal polyps') AND ('recurrence'/exp OR 'recurrence' OR 'relapse' OR 'edema'). In the Cochrane Library, we searched using "Chronic Rhinosinusitis" AND "Endoscopic Sinus Surgery" AND "Nasal Polyps" AND ("Recurrence" OR "Relapse" OR "Edema").

Additionally, we manually checked the reference lists of selected articles to identify any further relevant studies that might have been missed in the initial search.

Screening and Extraction

Articles were initially screened based on the relevance of their titles, with those deemed not pertinent were disqualified. Subsequently, the full texts and abstracts of the remaining papers were examined to include studies that met the predefined inclusion criteria. Exclusion criteria were applied rigorously to ensure that selected studies aligned with the systematic review's objectives, particularly focusing on outcomes related to post-ESS nasal polyp recurrence. Studies lacking detailed outcomes on recurrence or not specifically addressing this topic were excluded from further consideration. To maintain rigorous quality control, a double screening technique was employed. The first part of the screening technique analyzed the titles and abstracts of potential articles for initial eligibility, while the second part involved a comprehensive review of the entire texts in potentially relevant articles. This dual approach ensured that all significant articles were thoroughly evaluated for inclusion. Discrepancies between reviewers during the screening and extraction phases were resolved through discussion and consensus. In instances where consensus could not be reached, a third reviewer was consulted to adjudicate and finalize decisions. This process aimed to minimize bias and ensure that only studies meeting the systematic review's stringent inclusion criteria were included. Following the screening process, an organized extraction sheet was developed to systematically collect relevant data from the included studies. This extraction sheet captured key outcomes such as recurrence rates and identified risk factors, alongside baseline characteristics including study design, total sample size, and geographic source.

Quality Assessment

Quality assessment in this systematic review utilized the modified Newcastle-Ottawa Scale (NOS) for cohort studies to evaluate the methodological rigor of included studies across three primary domains: selection, comparability, and outcomes [11]. Each domain comprises specific criteria contributing to a maximum score of 10 stars, categorizing studies into poor (0-4), satisfactory (5-6), good (7-8), and very good (9-10) classifications based on their cumulative scores.

Under the selection domain, studies were scrutinized for the representativeness of their sampled populations, with emphasis on whether they adequately reflected the characteristics of the target population. The evaluation also considered the sufficiency of sample sizes to ensure robust statistical power and the handling of non-respondents to minimize potential bias. Ascertainment of exposure, such as the accuracy and reliability of methods used to identify interventions like ESS, was a critical component of this assessment.

In the comparability domain, studies were assessed based on their efforts to control for confounding factors between groups. Factors adjusted for included participant demographics (e.g., age), health behaviors (e.g., smoking), relevant comorbidities, surgical history, allergic profiles, presence of eosinophilic infiltration, and usage of systemic corticosteroids. This domain allowed for a maximum of two stars, reflecting the extent of adjustment made for these variables.

Regarding outcomes, rigorous evaluation centered on the methods employed to assess study outcomes and the robustness of statistical analyses conducted. Criteria included the validity and reliability of outcome measurement tools, appropriateness of statistical methods used, and adequacy of sample sizes for reliable outcome assessment. Furthermore, the reassessment of sample representativeness specifically concerning outcome measurement was considered to ensure consistency and reliability across studies.

The cumulative star ratings across these domains facilitated the overall classification of study quality, emphasizing studies with minimal bias and methodological robustness. This rigorous approach aimed to enhance the reliability and validity of findings within the systematic review.

Results

Search Results

**Figure 1:**
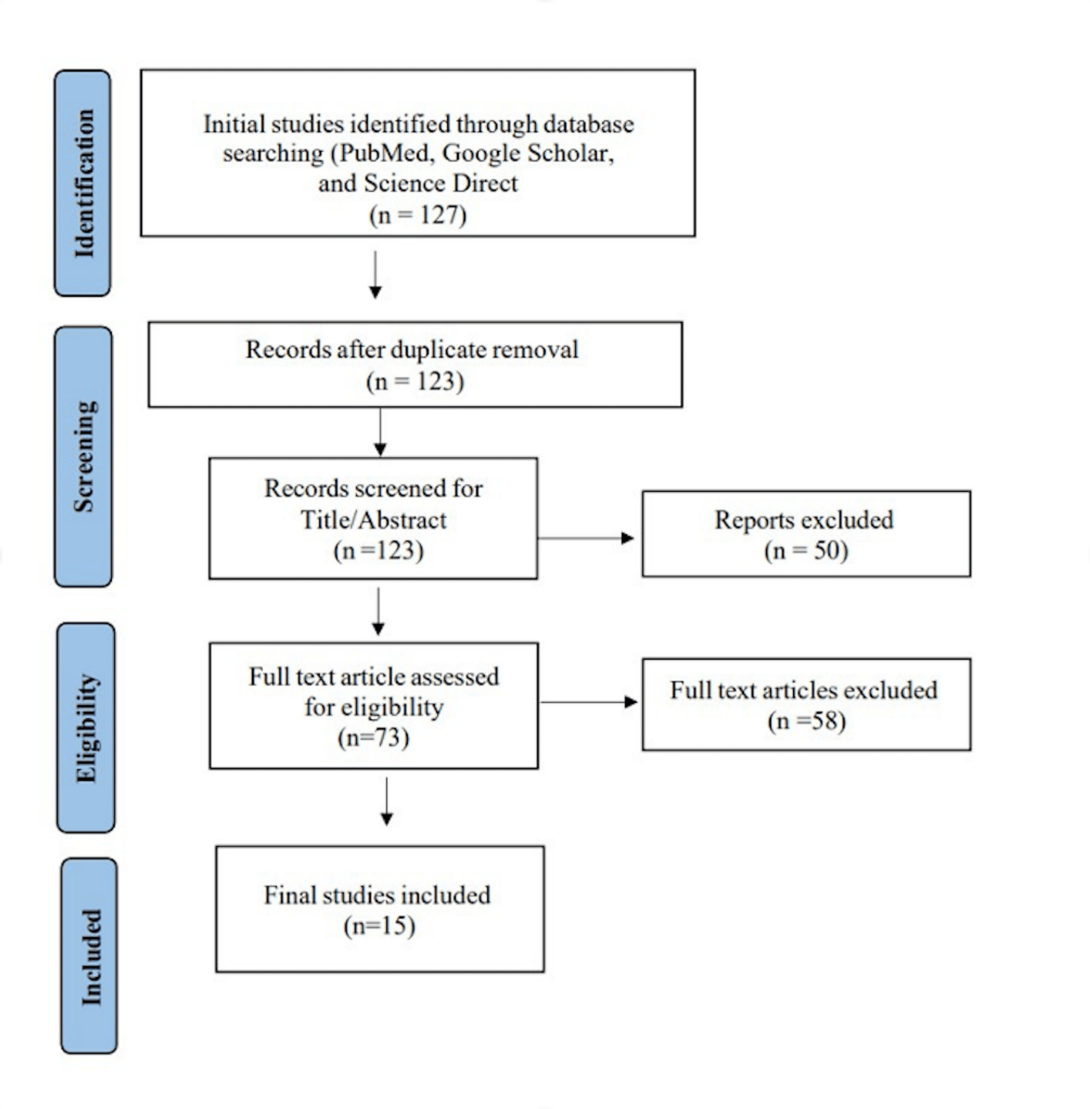
A PRISMA flow diagram outlining the study selection process PRISMA: Preferred Reporting Items for Systematic Reviews and Meta-Analyses

We were able to uncover a total of 127 citations using the previously specified search techniques, which were subsequently reduced to 123 after duplicates were eliminated. Only 73 citations remained after the title and abstract screening that qualified for the following stages. Only 15 articles met our inclusion and exclusion criteria after the full-text screening. Figure 1 [12] displays the thorough search and screening procedure.

Results of Quality Assessment

Most of the included research had good quality and a low risk of bias, according to our assessment of bias, while the remaining studies produced very good and satisfying results. None of the listed research studies produced unacceptable outcomes (Table 1).

**Table 1:** Summary of the results of bias assessment of the included studies using the modified Newcastle-Ottawa scale (NOS)

Study	Selection				Comparability		Outcome				Quality
	Representativeness of the sample	Sample size	Non- respondents	Ascertainment of the exposure	The subjects in different outcome groups are comparable	Assessment of outcome	Statistical analysis	Representativeness of the sample	Sample size	Total score	
												Telmesani et al. [13]	1	1	0	2	0	1	1	6	1	1	Satisfactory
Okushi et al. [14]	1	1	1	1	1	2	1	8	1	1	Good
Van Zele et al. [15]	1	0	1	1	1	2	1	7	1	0	Good
Vlaminck et al. [16]	1	0	0	1	1	2	1	6	1	0	Satisfactory
Brescia G et al. [17]	1	0	1	1	1	2	1	7	1	0	Good
DeConde et al. [18]	1	0	0	1	0	2	1	5	1	0	Satisfactory
Mohnsenh et al. [19]	1	1	0	1	1	2	1	7	1	1	Good
Meng et al. [20]	1	1	0	1	0	1	1	5	1	1	Satisfactory
Sella et al. [21]	1	1	1	1	1	2	1	8	1	1	Good
Rosati et al. [22]	1	1	0	1	0	2	1	6	1	1	Satisfactory
Salvador et al. [21]	1	1	0	1	2	1	1	7	1	1	Good
Beswick et al. [23]	1	1	1	1	0	2	1	7	1	1	Good
Bai et al. [24]	1	0	1	1	2	2	1	9	1	0	Very good
Brescia et al. [25]	1	0	1	1	1	1	1	6	1	0	Satisfactory
Riva et al. [26]	1	1	0	1	1	2	1	7	1	1	Good

Characteristics of the Studies Included

Finally, we examined 15 studies that satisfied the eligibility criteria of this investigation and are included in this systematic review. The studies enrolled participants between 2009 and 2022, including 2,515 ESS patients. The mean age of the included subjects ranged between 37.1 and 57.57 years. All of the included research investigations were observational studies, collecting data either prospectively or retrospectively. Regarding the countries from where the study participants were recruited, five studies were from Italy; three studies were from the USA; one study included participants from Canada as well; two studies were from Belgium; two were from Saudi Arabia; and one each from Brazil, China, Japan, and Portugal. The main characteristics of the included studies have been summarized in Table 2.

**Table 2:** Baseline characteristics of the included studies

Study	Country	Year	Study design	Total participants	Mean age (years)	Male gender %
Telmesani et al. [13]	Saudi Arabia	2009	Observational study	91	37.1 (13.2)	67.0%
Okushi et al. [14]	Japan	2012	Observational study	138	44	62.31%
Van Zele et al. [15]	Belgium	2014	Observational study	234	Median age, no recurrence: 47.55 (3.87); recurrence: 50.23 (4.33)	52.78%
Vlaminck et al. [16]	Belgium	2014	Observational study	221	47 (15.2)	56.11%
Brescia G et al. [17]	Italy	2015	Observational study	179	No recurrence: 51.8±15.3.; recurrence: 46.1±13.5	98.11%
DeConde et al. [18]	USA	2017	Observational study	363	49.8 ±14.5	60%
Mohnsenh et al. [19]	Saudi Arabia	2019	Observational study	257	No record	NR
Meng et al. [20]	China	2019	Observational study	230	No recurrence: 43.7 ± 7.1; recurrence: 44.7 ± 3.4	55.65%
Sella et al. [21]	Brazil	2020	Observational study	201	41±15	41.8%
Rosati et al. [22]	Italy	2020	Observational study	44	54.2	77.27%
Salvador et al. [21]	Portugal	2020	Observational study	132	43.4±11.5	62.1%
Beswick et al. [23]	USA, Canada	2021	Observational study	165	52.4±15.3	54%
Bai et al. [24]	USA	2022	Observational study	94	Median (IQR), 49.5 (41.8-60)	58.5%
Brescia et al. [25]	Italy	2022	Observational study	105	37.29±13.81	61.63%
Riva et al. [26]	Italy	2022	Observational study	61	57.57 ± 10.92	68.9%

Summary of the Study Outcomes

The outcomes of interest, including recurrence rates, risk factors, and follow-up periods, are presented in Table 3. The findings are categorized based on identified recurrence factors.

**Table 3:** Summary of the outcomes of the included studies in this review PEC: peripheral eosinophil count; REC: residual ethmoid cell count; ECP: eosinophilic cationic protein; ESS: endoscopic sinus surgery; E/M ratio: ratio of total ethmoid sinus scores for both sides and maxillary sinus score for both sides; CRS: chronic rhinosinusitis

Study	Recurrence rate	Risk factors/Predictors	Follow-up
Telmesani et al. [13]	50.5%	Eosinophilic mucin, IgE level	1 year
Okushi et al. [14]	-	PEC ≥ 9.5%, asthma, REC score of 4	-
Van Zele et al. [15]	24.4%	Asthma, aspirin intolerance, IgE level, specific IgE to* Staphylococcus aureus* enterotoxin level, ECP level, IL-5 expression	6.33 years
Vlaminck et al. [16]	39.6%	Tissue and airway mucus eosinophilia, eosinophilic mucin	3 years
Brescia G et al. [17]	13.40%	Eosinophilia, basophilia	36 months
DeConde et al. [18]	6-month: 35%, 12-month: 38%, 18-month: 40%	Prior ESS, preoperative polyposis severity	18 months
Mohnsenh et al. [19]	14.79%	Allergic fungal sinusitis	-
Meng et al. [20]	51.3%	Eosinophilia, asthma, surgical history onset CRS visual analog scores, anosmia score, E/M ratio, pre-ESS Lund-Kennedy score	24 months
Sella et al. [21]	32%	Asthma	12 years
Rosati et al. [22]	40.1%	Eosinophilia, asthma, aspirin intolerance, IL-5 expression	10 years
Salvador et al. [21]	34.1%	Asthma, aspirin intolerance, pre-ESS Lund-Kennedy score	3.2±1.5 years,
Beswick et al. [23]	43%	E/M ratio, type two (T-helper cell) profile	14.9 ± 4.8 months
Bai et al. [24]	39.4%	Eosinophilia, asthma, IL-5 expression, pre-ESS modified Lund Mackay score, anti–double-stranded DNA IgG	-
Brescia et al. [25]	Young adult, male: 29.0%; female: 11.6%; elderly male: 4.5%	Younger age, male sex	12 months
Riva et al. [26]	5-year: 30.29%, 10-year: 66.06%	Asthma, aspirin intolerance, eosinophilia, neutrophilia	126.49 ± 60.92 months

*Asthma as a Risk Factor for *Recurrence

Seven studies including Telmesani et al., Okushi et al., Van Zele et al., Sella et al., Salvador et al., Bai et al., and Riva et al. identified asthma as a significant risk factor for recurrence [14-16, 21, 24, 26, 27].

Eosinophilia and Recurrence

Six studies including Okushi et al., Vlaminck et al., Brescia et al., Meng et al., Rosati et al., and Riva et al. reported on the relationship between eosinophilia and the recurrence of chronic rhinosinusitis with nasal polyps [14, 16, 17, 20, 22, 26].

Aspirin Intake and Recurrence

Four studies examined the impact of aspirin intake on recurrence following ESS (Van Zele et al., Vlaminck et al., Rosati et al., Riva et al.) [15, 16, 22, 26].

Interleukin-5 (IL-5) Expression and Recurrence

Three studies by Van Zele et al., Bai et al., and Rosati et al. investigated IL-5 expression as a predictor of recurrence [15, 22, 24].

Interaction Effects

One study by Brescia et al. reported an interaction between patient age and gender with recurrence outcomes [25].

Follow-Up Periods

Studies included in this review reported follow-up periods ranging from six months to 12 years, influencing the assessment of recurrence rates and risk factors.

Detailed Insights: Study by Study

Eosinophilia, eosinophilic mucin (EM), and allergic fungal sinusitis: McHugh et al. conducted a systematic review and meta-analysis and observed that high tissue eosinophilia, as determined by eosinophil count per high-power field, demonstrated suitable diagnostic accuracy in predicting the occurrence of eosinophilic CRS [28]. Telmesani et al. studied the prevalence of allergic fungal sinusitis (AFS) among patients with nasal polyps in the eastern province of Saudi Arabia [13]. Their criteria for diagnosis was the positive histopathological presence of EM-containing hyphae. They found that 12.1% of the total CRS cases showed AFS, whereas the rest were typical CRSwNP presentations. There was a highly significant statistical difference in IgE levels between patients suffering from AFS and CRSwNP (p <.001), with AFS cases showing higher IgE levels (mean (SD) IgE level in IU/ml: 820.2 (752.8) vs. 242.1 381.3)). The recurrence rate in fungal sinusitis cases was 54.5%, and in non-AFS cases it was 50%. However, this was not found to be statistically significant. Out of the 91 patients, 66 (72.5%) did not have a history of asthma, while 25 (27.5%) suffered from asthma. Out of these 25 patients, only one has a diagnosis of AFS. Out of the 25 asthmatic patients, 24 suffered from non-fungal CRSwNP. However, the statistical analysis failed to reveal any relationship between asthma and any of the two types of CRSwNP (P >.05) [13].

Asthma and residual ethmoid cell score: Okushi et al. studied the association between residual ethmoid cell (REC) score and post-ESS recurrence of CRS [14]. It has been implicated that RECs when incompletely eliminated during surgery, make the patient susceptible to recurrent CRS. In the study, patients post ESS were categorized into two categories: those with complete improvement and those who had better outcomes after surgery were classified as the satisfactory outcome group, whereas patients who had no improvement at all or had undergone a worsening of symptoms post ESS were labeled as the poor outcome group. When these two groups were compared for differences in peripheral eosinophilic count (PEC), asthma prevalence rate, and total REC score, it was found that these three parameters were significantly higher in the poor outcome group as compared to the satisfactory outcome group. Moreover, a PEC of 9.5% or above, a diagnosis of asthma, and an overall REC score of 4 or above showed a significant correlation with a poor outcome [14].

Asthma, aspirin intolerance, and cytokine expression (IgE, specific IgE to *Staphylococcus aureus* enterotoxin, eosinophilic cationic protein (ECP), and IL-5): Van Zele et al. studied the cytokine profile in CRSwNPs that underwent FESS for the first time and recurrent CRSwNPs [15]. The tissue samples of CRSwNP patients without recurrence, patients who had a first revision surgery for recurrence, and patients who had a second revision surgery for recurrence were examined for the levels of IL-1 beta, IgE, specific IgE, IL-5, interferon (IFN) gamma, IL-6, IL-17, transforming growth factor (TGF) beta1, and myeloperoxidase. They found that the concentrations of IgE, specific IgE to *Staphylococcus aureus* enterotoxin, ECP, and IL-5 were significantly increased in recurrent versus nonrecurrent CRSwNPs at the time of the first surgery, while IL-17, IL-6, TGF-beta1, and IL-1 beta did not reveal any significant difference. IFN-gamma protein concentrations were significantly greater in nonrecurrent CRSwNPs. The odds ratio for recurrence of CRSwNPs was lowered to 0.029 if IFN-gamma was present in tissue homogenates. They also observed a significantly higher prevalence of asthma and intolerance to aspirin in recurrent CRSwNP cases that underwent revision surgery as compared to the nonrecurrent cases (p = 0.001 and p <0.0001, respectively) [15].

Eosinophilia: Vlaminck et al. studied the influence of eosinophils, EM, and fungal hyphae (FH) on the clinical outcome of ESS for CRS [16]. They found the presence of tissue eosinophils in 78.1% of cases of CRSwNP as compared with 42.4% of CRSsNP cases (p <0.001). Eosinophilic mucin was found to be present in the mucus airway secretions analysis in nearly 34% of cases and was higher in the CRSwNP group (20.0% vs. 52.1%; p <0.001). During a three-year follow-up period, a global recurrence rate of 22.2% was noted, which was nearly five times higher in the CRSwNP group as compared to the CRSsNP group (39.6% vs. 8.8%, p <0.001). In CRSwNP cases where tissue eosinophils were present, recurrence was present in 48% of cases, which was different as compared to cases without eosinophilic presence (p = 0.02). In CRSwNP cases where tissue eosinophils, EM, and FH were observed, a 72.7% recurrence rate was noted, while there was no recurrence in CRSsNP cases where these three elements were present. The analysis also revealed a poor outcome in the aspirin-sensitive group, where the total recurrence rate was 70%; however, 80% of aspirin-sensitive patients also had the abovementioned three elements present. The researchers in this study focused on eosinophilia over the traditional classifications of CRSwNP and CRSsNP. They reported that in eosinophilia-positive disease, 32.0% of the CRS cases relapsed, whereas in cases where eosinophilia was absent in both tissue and airway secretions, only 8.6% of cases faced recurrence (p <0.001). The authors concluded that eosinophilic presence in tissue or sinonasal mucosal secretions substantially raised the risk of recurrence in surgically treated CRSwNP patients [16].

Eosinophilia and basophilia: Brescia et al. (2015) studied the clinical, laboratory, and conventional pathology measures for accurately identifying CRSwNP patients at greater susceptibility to recurrence after functional ESS [17]. A significant relationship was noted between CRSwNP recurrence and serum basophil concentration (p = 0.03) and percentages (p = 0.02). The recurrence rate was greater for subjects with eosinophilia-positive CRSwNP (p = 0.01). In addition to univariate analysis, results from a multivariable logistic regression analysis revealed a similar result of individual prognostic importance with regard to recurrent CRSwNP disease [17].

Prior-ESS and preoperative polyposis intensity: DeConde et al. studied how prevalent recurrent nasal polyposis was in a follow-up period of up to 18 months after ESS with simultaneous medical therapy [18]. Of all the subjects, 67% of participants had graded postsurgical endoscopies, with a mean follow-up of 14.3 ± 7.0 months. Surgical intervention along with postoperative medical management significantly enhanced endoscopy total scores at six months (p <.001). Recurrent CRSwNP six months after ESS was 35%, compared to 38% after one year and 40% after 18 months. Multivariable analysis revealed both previous ESS (odds ratio (OR): 2.6, 95% confidence interval (CI): 1.5-4.6; p =.001) and worse preoperative polyp severity (OR: 1.4, 95% CI: 1.1-1.8; p =.016) as predisposing factors for recurrent polypi. The authors concluded that the recurrence of polyps was a common occurrence post-ESS, with a progressively controlled disease noted up to 18 months in 60%-70% of patients [18].

Allergic fungal sinusitis: Mohsenh et al. studied the risk factors and CT scan findings of recurrent post-ESS CRS subjects [19]. The global recurrence rate in the sample was 14.79%. Out of all the risk factors taken into consideration, including age, sex, airway, an inflammatory autoimmune condition, smoking, a subcategory of sinusitis, and anatomical variability, including those visualized via CT scans, they only found fungal sinusitis to be of statistical significance in relation to recurrent CRS. Anatomical or radiological findings using CT scanning post-surgically were thickened paranasal mucosa, nasal polyposis, septal deviation, and obliteration of the osteomeatal complex [19].

Eosinophilia, asthma, ethmoid to maxillary sinus opacification ratio, and prior-ESS: Meng et al. compared the use of CT scan findings and clinical findings in the prediction of recurrent CRSwNP [20]. These parameters included CT scores, exhaled nitric oxide level, PEC, and the extent of inflammatory cell infiltration in the sinonasal mucosa. In comparison to non-recurrent CRSwNP cases, cases with recurrence showed higher significance concerning parameters like surgical history onset and asthma, CRS visual analog scores, anosmia score, the ratio of total ethmoid sinus scores for both sides, and maxillary sinus score for both sides (EMR), Lund-Kennedy (LK) score, tissue eosinophil percentage, and tissue eosinophil absolute count. The EMR was noted to be highly accurate as a predictor for recurrent CRSwNP. The cut-off point of 2.5 for the EMR was identified as having the greatest predictive value for CRSwNP recurrence [20].

Eosinophilia, IL-5 expression, asthma, and aspirin intolerance: Rosati et al. studied the inflammatory profile in subjects with long-term CRSwNP recurrence and those without any recurrence [22]. They also compared the concentration of eosinophils, neutrophils, and lymphocytes in the inflammatory infiltrate as well as IL-5 and IL-8 expression in profiles showing long-term recurrence. The two measures that seemed to have a statistically significant influence on long-term recurrence were eosinophilia-positive infiltrate (p <0.05) and IL-5 expression (p <0.05). The presence of asthma and aspirin intolerance seemed to be more significantly common in recurrent cases, while allergy did not reveal a statistically significant difference between the recurrent and nonrecurrent groups [22].

Asthma: Sella et al. assessed the risk factors that potentially affected the recurrence risk in patients undergoing ESS in long-term follow-up [21]. The parameters assessed by the authors included nasal polyposis, asthma, nonsteroidal anti-inflammatory drug (NSAID)-exacerbated respiratory pathology, smoking habits, PEC, and atopy. They found that there was a higher likelihood of the need for revisional surgery in patients with CRSwNP than in those with CRSsNP (adjusted hazard ratio (HR), 2.02). The only factor that had a significant relationship with recurrence both in cases with CRSsNP (HR, 5.54) and with CRSwNP (HR, 3.27) was asthma. Even though eosinophilia was not associated with a greater possibility of recurrent CRS, its existence affected the outcome of CRSwNP in comparison with CRSsNP and the effect of asthma among cases with CRSwNP. The authors concluded that the prognosis in cases with CRSwNP was poorer than that in cases with CRSsNP. Asthma was the only factor that raised the probability of recurrent CRS in individuals with either CRSsNP or CRSwNP [21].

Asthma, aspirin intolerance, and preoperative LK score: Salvador et al. assessed the surgical outcomes of ESS in CRSwNP treatment and evaluated the independent risk factors for recurrent disease and surgical revision [27]. Asthma was the most frequent comorbid condition (39.4%), and aspirin-exacerbated respiratory disease (AERD) was observed in 9.8% of the subjects. A recurrence rate of 34.1% was noted, and 9% of patients required a revision ESS procedure. Multivariable analysis found independent measures of recurrent disease (95% CI): a history of asthma (OR = 8.81, CI 3.87-20.03; p <0.001) and a high Lund-Mackay (LM) score (17-24) (OR = 5.85, CI 2.73-12.51; p = 0.001). Revision surgery was associated with an extreme endoscopic LK score at presentation (OR = 4.05, CI 1.91-8.01, p = 0.001). The authors concluded that CRS with sinonasal polyposis displayed a more severe predisposition for recurrent disease after ESS. Asthma, severely opacified sinuses, and a high endoscopic score represented a poor prognosis and a more aggressive disease course [27].

Ethmoid to maxillary sinus opacification ratio and type 2 T-helper cell profile: Beswick et al. studied the influence of the EMR during presurgical scanning on surgical outcomes pertaining to nasal polyposis and postoperative olfaction [23]. The subjects comprised a North American/type 2 T-helper cell (type 2) profile population. The 22-item SNOT-22, Brief Smell Identification Test (BSIT), and LK endoscopic scores were obtained pre-and postoperatively. Patients were stratified according to increasing EMRs based on LM scores. On average, significant within-subject postsurgical improvement was noted in all patients for SNOT-22 total and domain scores and BSIT results (p ≤ 0.019). Pre-surgically, an elevated EMR was correlated with worse BSIT scores (r = −0.343, p < 0.001). Post-surgically, an elevated EMR correlated with improved scores on the BSIT (r = 0.284, p = 0.002) but failed to show a correlation with SNOT-22 improvement or polyposis recurrence. An elevated E/M ratio was associated with a higher chance of reporting a minimal clinically meaningful difference in BSIT scores (χ2 = 9.96, p = 0.041). Increased EMRs were found to be related to poorer baseline olfaction and an elevated likelihood of getting a therapeutically important postsurgical improvement in olfaction in these North American subjects with CRSwNP. Elevated EMRs did not predict postsurgical alterations in SNOT-22 measures or polyposis recurrence. This led the authors to conclude that prognostic factors may vary according to geography and generalized inflammatory profiles (type 2 vs. non-type 2) in cases with CRS [23].

Eosinophilia, asthma, IL-5, preoperative modified LM (MLM) score, and anti-double-stranded DNA IgG: Bai et al. assessed the impact of recurrent nasal polyposis on measures of CRSwNP severity post-ESS and attempted to quantify the prognostic value of different clinical parameters [24]. They found recurrent polyposis in 39.4% of patients, despite significant improvements in MLM and SNOT-22 scores (p<0.001). Recurrent polyps were significantly related to poorer post-ESS MLM scores, modified LK scores, and SNOT-22 scores. The authors proposed that a combined model comprised of eosinophil cationic protein, IL-5, pre-ESS MLM score, asthma, and anti-double-stranded DNA IgG could accurately predict polyp recurrence. Using a random forest analysis, they detected and validated the five variables as the strongest predictors of recurrent nasal polyposis in CRS [24].

Younger age and male sex: Brescia et al. (2021) compared the clinical, laboratory, pathological, and prognostic features of ESS-treated CRSwNP in male vs. female patients stratified according to age (young-adult (20 years ≤ age ≤ 40 years) and elderly (age ≥ 65 years)) [25]. They found that the recurrence rate was related to the age and gender combination (p = 0.0165). Young adult males' recurrence rate (29.0%) was higher than that of young adult females (11.6%) and older males (4.5%). Allergy resulted in a relationship with age and sex combination (p = 0.0158). Young adult males' allergy rate (50.0%) was greater than older males' (29.5%) and older females' (13%). Moreover, the allergy rate was greater in young adult females (41.9%) than in older females. They concluded that it was possible that gender and age variables interacted in the causation of recurrent polyposis post-ESS [25].

Asthma, aspirin intolerance, eosinophilia, and neutrophilia: Riva et al. assessed the CRSwNP recurrence risk following a long-term follow-up (up to 20 years), as well as the role of nasal cytology. Five- and 10-year recurrence rates were 30.29% and 66.06%, respectively [26]. Median recurrence-free survival was 106 months. Asthma and AERD represented predictive factors for multiple recurrences (p<0.05). Intranasal steroids were the primary treatment to avoid treatment relapse (p<0.05). Subjects with normal cytology at follow-up assessment had a decreased likelihood of first recurrence within 10 years (59% of cases) in comparison to neutrophil or eosinophil infiltrate (100% and 88% of cases, respectively) (p<0.05). The authors concluded that CRSwNP had a high recurrence risk, also more than 10 years after surgery, and that nasal cytology holds importance in identifying patients with a greater susceptibility for early recurrence [26].

Discussion

Chronic rhinosinusitis with nasal polyps is a chronic inflammatory disease that can impose a significant burden on patients, healthcare systems, and society. The burden of CRSwNP can be categorized into several domains, including economic, social, and health-related. Chronic rhinosinusitis with nasal polyposis can impact a person's social life, causing social isolation and decreased participation in activities. The symptoms of CRSwNP, such as facial pain, nasal congestion, and hyposmia/anosmia, can lead to embarrassment, frustration, and avoidance of social situations. CRSwNP can significantly impact a person's physical and emotional well-being. The symptoms of CRSwNP can cause physical discomfort, decrease sleep quality, and lead to psychological distress. In addition, CRSwNP has been associated with an increased risk of developing asthma and other comorbidities, which can further increase the burden on patients.

Predictors of Recurrence of CRSwNP

The studies included in this systematic review consistently identify several predictors associated with the recurrence of CRSwNP. These predictors include eosinophilia, asthma, AERD, preoperative polyp severity, residual ethmoid cells, specific cytokine expressions (e.g., IL-5), prior ESS, and demographic factors such as age and sex. Understanding these predictors and their underlying mechanisms is crucial for developing targeted interventions and improving patient outcomes.

Eosinophilia: Eosinophilia is a robust predictor of CRSwNP recurrence. Eosinophils play a pivotal role in tissue repair and immune defense, particularly against helminths. Initially, CRSwNP was often viewed as primarily eosinophilic, characterized by eosinophil-mediated mucosal damage. However, it became evident that non-eosinophilic forms also exist, challenging this notion [29]. Studies segregating CRSwNP and CRSsNP samples revealed higher tissue eosinophilia in polyps, suggesting a close association independent of atopy. Yet, this association is less pronounced in Asian populations and a minority of Western polyps compared to most Caucasian cases.

Despite eosinophils not being indispensable for CRS or nasal polyposis, they serve as biomarkers for severe, treatment-resistant disease. Their recruitment, activation, and survival in CRS are driven by epithelial cytokines, proteases, and Type 2 cytokines, primarily originating from ILC2 and Th2 cells. Eosinophilic activity, including degranulation, contributes to tissue edema, epithelial damage, and potentially fibrosis, akin to mechanisms observed in asthma. Moreover, eosinophils aid in host defense by forming extracellular traps against bacteria, albeit at the cost of compromising barrier integrity.

Therapeutically, eosinophils respond to steroids, explaining the efficacy of glucocorticoids in CRS. Targeted therapies like anti-IL-5 have shown promise in reducing eosinophilic inflammation and improving clinical outcomes in CRSwNP patients with a Type 2 cytokine profile. However, not all patients respond equally to these treatments, highlighting the complex pathophysiology where eosinophils may represent only part of the Type 2 CRS spectrum.

Asthma: Asthma exacerbates sinonasal inflammation through a hyperreactive airway state, further aggravating nasal polyposis and leading to recurrence. Asthma is strongly associated with CRSwNP recurrence. Studies by Okushi et al. [14], Van Zele et al. [15], and Salvador et al. [27] found that patients with these conditions had higher recurrence rates post-ESS. Asthma represents a significant predictor of recurrence in CRSwNP, reflecting a complex interplay between upper and lower airway inflammatory diseases. Numerous studies have consistently demonstrated a heightened prevalence of asthma among CRSwNP patients compared to the general population, emphasizing asthma's role as a comorbid condition that influences CRSwNP pathogenesis and clinical outcomes [30].

The association between asthma and CRSwNP is multifaceted. Asthma, characterized by chronic airway inflammation, shares underlying immunological mechanisms with CRSwNP, such as Type 2 inflammatory responses involving cytokines like IL-4, IL-5, and IL-13. These cytokines contribute to tissue eosinophilia and remodeling processes observed in both asthma and nasal polyposis, thereby promoting a chronic inflammatory milieu that sustains disease recurrence and severity.

Beyond shared immunological pathways, epidemiological studies consistently highlight asthma as a risk factor for developing CRSwNP. The prevalence rates of CRSwNP are markedly elevated among asthma patients, suggesting a bidirectional relationship where asthma exacerbates nasal inflammation and polyposis formation, while nasal symptoms and polyp growth adversely affect asthma control and exacerbation rates. Chen et al. conducted a study in Taiwan on patients newly diagnosed with asthma to analyze the incidence of CRS within this population [31]. After adjusting for variables such as gender, age, and other medical conditions, they found that asthma is an independent predictor of CRS, both with and without nasal polyps (OR: 2.58 for CRS without nasal polyps).

Clinically, the presence of asthma in CRSwNP patients often correlates with more severe disease manifestations, including higher nasal symptom scores, greater polyp burden, and increased rates of surgical intervention. Moreover, the chronic inflammatory state in asthmatic CRSwNP patients may contribute to treatment resistance and higher rates of disease recurrence post-surgery, necessitating vigilant follow-up and tailored therapeutic approaches. This mutual exacerbation underscores the importance of comprehensive management strategies that address both upper and lower airway diseases concurrently to achieve optimal clinical outcomes.

Aspirin-exacerbated respiratory disease: Aspirin-exacerbated respiratory disease, also known as Samter's triad, represents a distinct phenotype within CRSwNP, characterized by a triad of asthma, nasal polyposis, and hypersensitivity to NSAIDs such as aspirin. This condition poses unique challenges due to its refractory nature and high propensity for disease recurrence. In our systematic review, studies by Okushi et al. [14], Van Zele et al. [15], and Salvador et al. [27] found that patients with these conditions had higher recurrence rates post-ESS.

Aspirin-exacerbated respiratory disease, or more generally, NSAID-exacerbated respiratory disease (N-ERD), represents a chronic inflammatory disorder of the respiratory tract characterized by eosinophilic inflammation in patients with asthma and/or CRSwNP, exacerbated by NSAIDs, particularly aspirin. The pathogenesis of N-ERD involves dysregulated eicosanoid synthesis, leading to eosinophilic inflammation of the nasal and sinus membranes and increased production of leukotrienes, which is further amplified by cyclooxygenase (COX)-1 inhibition by NSAIDs like aspirin. Respiratory reactions to NSAIDs are reported more frequently in individuals with concurrent CRS and asthma symptoms, although not necessarily allergic rhinitis. In a UK analysis, self-reported aspirin sensitivity was significantly higher among CRSwNP patients (9.6%) and those with AFS (40%), compared to controls (2.3%) [32]. Patients with N-ERD tend to experience more severe upper respiratory symptoms, greater sinus opacification on CT scans, and higher rates of nasal polyp recurrence following surgery compared to NSAID-tolerant CRSwNP patients. Diagnosis of N-ERD primarily relies on patient history, with aspirin provocation tests employed when clinical history is unclear. These patients typically undergo more frequent sinus surgeries and present at a younger age for their initial surgery compared to CRSwNP patients without NSAID sensitivity. The clinical management of N-ERD necessitates a comprehensive approach addressing both upper and lower airway inflammation, often involving surgical interventions such as ESS to alleviate symptoms and improve quality of life. However, due to the chronic and refractory nature of the disease, ongoing monitoring and individualized treatment strategies, including biologic agents targeting inflammatory pathways, are crucial to mitigate disease recurrence and improve long-term outcomes.

Cytokine expressions: Elevated levels of IL-5, IgE, and specific IgE to *Staphylococcus aureus* enterotoxins are implicated in CRSwNP recurrence. Van Zele et al. demonstrated significantly increased cytokine levels in recurrent cases, suggesting their role in sustaining eosinophilic inflammation [15]. IL-5 is critical for eosinophil survival, recruitment, and activation, creating a persistent inflammatory environment that fosters recurrence.

Cytokine expression profiles play a pivotal role in the pathogenesis and recurrence of nasal polyposis in CRS. The intricate interplay of cytokines, particularly those associated with Type 2 inflammation, influences disease severity, recurrence rates, and treatment outcomes in CRS patients with nasal polyposis. Type 2 cytokines, including interleukins such as IL-4, IL-5, and IL-13, are prominently expressed in CRSwNP. These cytokines orchestrate eosinophilic inflammation, mucus hypersecretion, and tissue remodeling within the sinonasal mucosa. Elevated levels of IL-5, for instance, are associated with eosinophil recruitment and activation, contributing to polyp formation and persistence. Studies have consistently demonstrated that patients with higher baseline levels of Type 2 cytokines exhibit more severe CRSwNP phenotypes and are prone to disease recurrence following treatment interventions [33]. Persistent elevation of IL-5, IL-13, and other pro-inflammatory cytokines correlates with increased polyp size, greater symptom burden, and reduced treatment responsiveness. Furthermore, the balance between Type 1 and Type 2 cytokines is crucial in determining disease course and recurrence risk in CRSwNP. Imbalance towards Type 2 cytokines not only sustains chronic inflammation but also compromises mucosal barrier function, perpetuating a cycle of tissue damage and repair.

Therapeutic strategies targeting cytokine pathways have shown promise in managing CRSwNP. Biologic agents, such as monoclonal antibodies against IL-5 (e.g., mepolizumab) or IL-4/IL-13 (e.g., dupilumab), aim to modulate Type 2 inflammatory responses and improve clinical outcomes. These treatments not only reduce polyp size and nasal congestion but also mitigate recurrence rates by addressing underlying inflammatory mechanisms. In clinical practice, monitoring cytokine profiles may serve as a biomarker for disease severity and recurrence risk in CRSwNP patients.

Preoperative polyp severity: A more severe preoperative polyp burden indicates a more aggressive disease phenotype, likely to recur despite surgical intervention. Higher preoperative polyp severity post-ESS is predictive of recurrence, as noted by DeConde et al. [18] and Okushi et al. [14].

This assessment typically involves standardized scoring systems such as the LK score or the Polyp Score, which evaluate the extent and chronicity of sinonasal inflammation and polypoid disease. High preoperative polyp severity scores indicate more extensive inflammatory involvement and chronic disease burden. Patients presenting with severe polypoid disease often experience more challenging surgical outcomes and higher rates of postoperative recurrence. The severity of polypoid disease reflects the underlying inflammatory milieu within the sinonasal mucosa, which contributes significantly to disease persistence and recurrence. Stratifying patients based on preoperative polyp severity allows for personalized treatment planning and management. It helps guide surgical decision-making, including the extent of surgical intervention required to achieve disease control and minimize recurrence. Advances in imaging techniques, such as high-resolution CT scans, enhance the accuracy of preoperative assessments by providing detailed anatomical information and aiding in the identification of disease extent.

Residual ethmoid cells: Residual ethmoid cells post ESS are predictive of recurrence, as noted by DeConde et al. [18] and Okushi et al. [14]. The presence of residual ethmoid cells indicates incomplete disease clearance during surgery, influenced by anatomical variations, disease severity, and surgical approach. These residual cells can harbor inflammatory foci, serving as potential niduses for ongoing mucosal irritation, polyp regrowth, and recurrence of symptoms postoperatively. Managing residual ethmoid cells is critical in preventing disease recurrence. It necessitates comprehensive surgical planning and meticulous intraoperative execution to maximize disease clearance and minimize residual inflammation. Advances in imaging modalities, such as endoscopic assessments and intraoperative navigation systems, aid in identifying and addressing residual disease burden effectively. Tailoring postoperative management strategies, including pharmacological therapies and regular follow-up, is essential to mitigate residual inflammation and prevent recurrence. Monitoring of residual ethmoid cells through postoperative imaging allows for early detection of disease recurrence and prompt intervention, thereby optimizing long-term disease control. Further research is warranted to explore innovative strategies for reducing residual ethmoid cells and enhancing surgical outcomes in CRS patients with nasal polyposis. Integrated approaches combining surgical precision with advanced imaging techniques and biomarker assessments hold the potential to improve patient outcomes and quality of life by minimizing the burden of disease recurrence.

Prior ESS: A history of ESS is a significant risk factor for recurrence. Persistent inflammatory pathways not fully addressed by initial surgery could contribute to ongoing polyp formation. DeConde et al. found that patients requiring multiple surgeries had a higher likelihood of recurrence, suggesting that repeated surgical intervention may indicate refractory disease [18]. While ESS initially aims to alleviate symptoms and reduce polyp burden, recurrence rates vary due to factors such as disease severity, surgical technique, and patient characteristics. Patients with a history of ESS often present with complex disease profiles characterized by severe inflammation and recurrent polyp formation. Despite initial surgical success, a significant proportion experience disease recurrence over time. Incomplete disease resolution plays a pivotal role in recurrence post-ESS. Residual disease, including missed ethmoid cells or frontal recess disease, contributes to persistent inflammation and promotes polyp regrowth. Chronic mucosal inflammation persists despite surgery, driven by immune dysregulation and cytokine-mediated pathways. This inflammatory environment fosters disease relapse, highlighting the challenge of achieving long-term disease control in these patients [33]. Moreover, postoperative tissue remodeling and scarring further complicate management. Surgical healing processes, including fibrosis and anatomical alterations, can predispose to recurrent polyp formation and symptomatic recurrence. Management strategies must thus address these complexities through comprehensive preoperative assessment, refined surgical techniques, and optimized postoperative care. Effective preoperative assessment is crucial, utilizing detailed evaluations of disease severity, imaging studies, and endoscopic findings to inform surgical planning. Advancements in surgical techniques, such as image-guided navigation and minimally invasive approaches, enhance precision and improve outcomes by ensuring complete disease clearance. Vigilant postoperative monitoring and timely intervention are essential to detect early signs of recurrence and adjust therapeutic strategies accordingly. Future research should focus on predictive models and biomarkers to identify high-risk patients and elucidate underlying pathophysiological mechanisms. Investigating genetic predispositions and inflammatory markers may provide insights into personalized treatment approaches. Ultimately, optimizing management strategies for CRS patients with prior ESS is essential to achieve durable disease control and improve long-term patient outcomes.

Demographic factors: Demographic factors might impact hormonal influences or immune response differences, affecting the inflammatory profile and recurrence risk. Age, gender, ethnicity, and socioeconomic status contribute to variations in disease presentation and recurrence rates among CRS patients. Brescia et al. identified younger age and male sex as associated with higher recurrence rates [25]. Age is a notable demographic factor impacting CRS recurrence. Older patients often present with more severe disease and higher rates of comorbidities, which can complicate surgical outcomes and increase the likelihood of polyp recurrence [34]. Conversely, younger patients may exhibit different disease phenotypes, potentially influenced by immune maturation and environmental exposures. Gender differences also influence CRS recurrence rates, with some studies suggesting varying disease severity and treatment responses between males and females. Hormonal influences, immune responses, and anatomical differences in sinonasal anatomy contribute to these disparities, highlighting the need for tailored treatment approaches based on gender-specific considerations.

Ethnicity and genetic predispositions play a crucial role in CRS pathophysiology and recurrence risk. Variations in inflammatory responses and genetic polymorphisms contribute to ethnic disparities in disease prevalence and severity. Studies indicate that a familial tendency significantly influences the likelihood of developing CRSwNP, highlighting the importance of genetic factors in the disease's pathophysiology and recurrence rates. Recent research by Oakley et al. has shed light on the heritability of CRSwNP. In a study involving 1,638 patients with CRSwNP and 24,200 patients with CRSsNP, it was found that first-degree relatives of CRSwNP patients are 4.1 times more likely to develop CRSwNP themselves [35]. This risk extends to CRSsNP, with a 2.4 times higher likelihood among first-degree relatives. Notably, the study also suggested an environmental component, as spouses of affected individuals were twice as likely to develop CRSsNP, underscoring the interplay between genetic and environmental factors in disease manifestation. Complementary findings from Sweden further support the genetic link. Screening of relatives of patients with nasal polyposis revealed that 13.4% had nasal polyps, compared to only 2.7% in a control group randomly selected from the general population [36]. This translates to a relative risk of 4.9 for first-degree relatives, reinforcing the significant heritable component in CRSwNP. These findings have crucial implications for the clinical management of CRSwNP. Understanding the genetic predisposition can aid in identifying individuals at higher risk for recurrence, enabling more personalized and proactive treatment approaches. Patients with a family history of CRSwNP may benefit from closer monitoring and early intervention strategies to mitigate the risk of recurrence. Furthermore, the identification of genetic markers associated with CRSwNP recurrence could pave the way for targeted therapies. Genetic screening could become an integral part of the diagnostic process, allowing for the stratification of patients based on their genetic risk and tailoring of treatment protocols accordingly.

Additionally, socioeconomic factors, including access to healthcare, environmental exposures, and lifestyle habits, impact CRS recurrence rates. Socioeconomic status influences treatment adherence, healthcare utilization patterns, and access to advanced therapies, which can significantly affect disease control and recurrence risk among different patient populations. Furthermore, demographic factors interact with environmental and lifestyle factors to shape CRS recurrence. Environmental factors play a pivotal role in the recurrence of CRSwNP, complementing the genetic predisposition of the disease. While genetic studies indicate a significant heritable component, the influence of environmental factors cannot be overlooked, as they significantly affect the likelihood of disease recurrence. A large database study has highlighted the significant familial risk associated with pediatric CRS, suggesting a strong genetic component [37]. However, studies on monozygotic twins have demonstrated that both siblings do not always develop nasal polyps, underscoring the critical role of environmental factors in the manifestation and recurrence of CRSwNP. This indicates that while genetics lay the foundation, environmental exposures and lifestyle choices significantly shape disease outcomes.

Exposure to allergens is one of the most critical environmental factors influencing CRSwNP recurrence. Allergens such as pollen, dust mites, and animal dander can trigger inflammatory responses in the nasal mucosa, exacerbating symptoms and leading to polyp regrowth. Patients with CRSwNP often exhibit heightened sensitivity to these allergens, making environmental control measures essential in managing the disease. Air pollution is another significant environmental factor contributing to CRSwNP recurrence. Pollutants such as particulate matter, ozone, and nitrogen dioxide have been linked to respiratory inflammation and increased susceptibility to sinus infections [38]. Studies have shown that individuals living in areas with high levels of air pollution are at greater risk of developing CRSwNP and experiencing recurrent symptoms. Reducing exposure to polluted environments and advocating for cleaner air policies can mitigate the impact of air pollution on CRSwNP recurrence.

Occupational exposure to dust emerges as a significant factor influencing the recurrence of CRSwNP, as highlighted by Veloso-Teles et al. [39]. Their study, consistent with findings by Hox et al., revealed a high prevalence (60%) of reported exposure to organic and inorganic dust among patients with CRSwNP [40]. This exposure significantly increased the risk of recurrence, with exposed individuals showing a 38 times greater likelihood of recurrence compared to non-exposed individuals (odds ratio 38 (95% CI, 4-395); p < 0.001). Moreover, similar to Hox et al., Veloso-Teles et al. identified a predominant exposure to low molecular weight (LMW) agents (90.6%) among patients with relevant occupational exposures [39]. Low molecular weight agents are known for inducing non-IgE-mediated mechanisms in airway inflammation, which correlates with poorer surgical outcomes for CRSwNP. Specifically, non-IgE-mediated asthma, often associated with LMW agents, significantly impacted recurrence rates, increasing the likelihood approximately ninefold (odds ratio 8.7 (95% CI, 2-46); p < 0.012). This suggests that occupational exposures, particularly to LMW agents, may serve as markers of disease severity in CRSwNP. These findings underscore the public health implications of occupational dust exposure in CRSwNP, aligning with recommendations from previous studies. Protective measures such as wearing masks during work and ensuring the functionality of air-dust filters and exhausting systems are crucial to mitigate disease recurrence risks. Prospective studies in the future will better help establish causal relationships between occupational dust exposure and CRSwNP recurrence, alongside investigations into the therapeutic impact of different corticosteroid delivery methods post ESS. Such efforts promise to enhance treatment outcomes and inform targeted interventions in clinical practice.

Further among environmental factors, infections, particularly viral respiratory infections, are known to exacerbate CRSwNP and contribute to recurrence. Firstly, viral infections play a pivotal role in the pathogenesis and exacerbation of CRSwNP, influencing disease onset and progression through complex immunological mechanisms. Respiratory viruses such as rhinovirus, influenza virus, and respiratory syncytial virus (RSV) are known triggers, initiating acute inflammatory responses in the upper respiratory tract that extend into the sinuses [41]. This initial inflammatory cascade involves the release of pro-inflammatory cytokines and chemokines, which recruit neutrophils, eosinophils, and other immune cells to the nasal and sinus mucosa. The persistence of viral-induced inflammation can lead to chronic mucosal changes characteristic of CRSwNP, including epithelial barrier dysfunction, glandular hyperplasia, and fibrosis. Viral infections also disrupt mucociliary clearance, impairing sinus drainage and promoting bacterial colonization, further complicating the disease course [42]. Clinically, viral infections are associated with acute exacerbations of CRSwNP symptoms, exacerbating nasal obstruction, rhinorrhea, facial pain or pressure, and olfactory dysfunction [43]. These exacerbations often require intensified medical management and may necessitate surgical intervention in severe or refractory cases. Preventive measures, such as vaccination against common respiratory viruses and practicing good hygiene, can help reduce the frequency of infections and subsequent CRSwNP recurrence.

Lifestyle factors, including smoking and diet, also influence CRSwNP recurrence. Smoking is a well-established risk factor for chronic respiratory diseases and has been shown to worsen the symptoms and recurrence of CRSwNP [44]. Encouraging smoking cessation is a crucial component of managing CRSwNP. Stress and psychological factors have also been implicated in the recurrence of CRSwNP. Chronic stress can impair immune function and increase susceptibility to infections and inflammation, leading to polyp regrowth. Stress management techniques, including mindfulness, exercise, and counseling, can be beneficial in mitigating the impact of stress on CRSwNP. Therefore, while genetic predisposition is a significant factor in the recurrence of CRSwNP, environmental influences play a crucial role in shaping disease outcomes. Addressing environmental factors through lifestyle modifications, exposure control, and preventive measures is essential in managing and reducing the recurrence of CRSwNP.

The ethmoid-to-maxillary sinus opacification ratio: This ratio quantifies and reflects the overall inflammatory burden in the ethmoid and maxillary sinuses, providing insight into the severity and distribution of sinonasal inflammation. Meng et al. [20] and Beswick et al. [23] identified the EMR as a predictor of recurrence. A higher EMR indicates extensive ethmoid involvement relative to the maxillary sinus, challenging to clear surgically, and thus prone to recurrence [45].

The ethmoid sinuses are centrally located within the sinonasal cavity and are often the primary sites of inflammation in CRS with nasal polyposis. The intricate anatomy and the dense concentration of inflammatory cells in the ethmoid sinuses contribute to a more challenging disease course and a higher likelihood of recurrence post surgery. Studies have demonstrated that patients with a higher preoperative EMR are at an increased risk for recurrence of nasal polyps following ESS [46]. The ethmoid sinuses' central role in CRS pathophysiology means that extensive ethmoid involvement often reflects a more aggressive and recalcitrant disease phenotype [47]. This extensive inflammation can persist despite surgical intervention, leading to recurrent polyp formation. Additionally, the EMR can serve as a guide for surgical planning and postoperative management [48]. Surgeons can use this ratio to identify patients who may benefit from more aggressive or extended surgical approaches to address extensive ethmoid disease. Postoperatively, a higher EMR may indicate the need for closer monitoring and more intensive medical therapy to mitigate the risk of recurrence. The predictive value of the EMR also underscores the importance of comprehensive preoperative imaging in CRS management. High-resolution CT scans are essential for accurately assessing the extent of disease in the ethmoid and maxillary sinuses, enabling precise calculation of the EMR [47]. This detailed imaging assessment allows for a tailored surgical approach and informs postoperative care strategies aimed at reducing recurrence rates.

Therefore, the mechanisms driving CRSwNP recurrence are multifactorial. Eosinophils, through the release of cytotoxic granules and cytokines, perpetuate mucosal inflammation. Asthma and AERD contribute through systemic inflammatory pathways involving leukotrienes. Cytokines like IL-5 enhance eosinophilic activity, exacerbating inflammation. Anatomical factors, such as residual ethmoid cells, provide a structural basis for polyp regrowth. Previous surgeries may indicate incomplete resolution of the disease process, while demographic factors may influence the immunologic landscape. These predictors interact to create a persistent inflammatory milieu, fostering recurrence.

Preventing recurrent disease is crucial to enhancing health-related quality of life and patient satisfaction. The continued use of intranasal corticosteroids post-surgery has demonstrated an improvement in postoperative endoscopic scores across all CRS patients and specifically reduces the risk of recurrence in those with CRSwNP [49]. Strategies to ensure medication adherence, such as leveraging digital technology, can help to further prevent recurrence. Additionally, other methods to ensure consistent application of postoperative medication, such as drug-eluting stents, may address compliance issues [50]. Since a few studies have indicated that ongoing occupational exposure to irritants might increase the risk of recurrence, addressing any factors believed to contribute to the underlying etiology of CRS in each patient, where possible, is essential to minimize the risk of recurrence. By recognizing these predictors and understanding their mechanisms, clinicians can better identify high-risk patients and tailor treatment plans to mitigate the risk of recurrence, such as through more aggressive medical management or targeted surgical techniques.

Recommendations for Future Research

This systematic review underscores several key factors influencing recurrence rates following ESS for CRSwNP. Moving forward, several avenues for future research emerge to further elucidate these complexities and enhance treatment outcomes. Longitudinal studies are essential to track patients over extended periods post-surgery, elucidating the natural history of CRSwNP recurrence. These studies can identify late-emerging risk factors and inform personalized treatment strategies and patient counseling regarding long-term prognosis. Randomized controlled trials (RCTs) are warranted to evaluate novel interventions aimed at reducing recurrence rates. Trials should explore optimized postoperative care protocols, biological therapies targeting inflammatory pathways specific to CRSwNP, and patient-tailored treatment algorithms based on identified risk profiles. Comparative effectiveness research could further delineate the benefits of surgical versus medical management approaches in preventing recurrence. Collaborative multicenter studies are crucial to enhance generalizability across diverse patient populations and healthcare settings. By pooling data, these studies can develop evidence-based guidelines for managing CRSwNP recurrence, addressing variations in treatment outcomes and patient responses. Genetic and biomarker studies offer promising avenues for personalized medicine in CRSwNP. Investigating genetic predispositions and biomarkers associated with disease recurrence could enable early identification of high-risk patients and facilitate targeted interventions to mitigate recurrence risk. Health economic evaluations are needed to assess the cost-effectiveness of preventive strategies versus reactive treatments for recurrent CRSwNP. Understanding the economic burden and cost implications of different management approaches will guide resource allocation and healthcare policy. Patient-centered outcomes research should prioritize assessments of the quality of life, treatment satisfaction, and patient-reported outcomes specific to CRSwNP recurrence. These metrics provide valuable insights into the holistic impact of recurrence on patients' lives and treatment preferences, informing shared decision-making between healthcare providers and patients.

Potential Risk of Bias Within and Across Studies

In evaluating CRSwNP recurrence following ESS, it is critical to consider the potential biases within and across the included studies. Understanding these biases is essential for interpreting the results accurately and for informing future research directions.

Selection bias is a prominent concern in studies by Telmesani [13], Okushi et al. [14], and Van Zele et al. [15], where specific patient populations were selected based on particular criteria that may not be representative of the broader CRSwNP patient population. For instance, Telmesani selected patients based on their ability to undergo regular follow-ups, potentially excluding those with more severe disease or comorbidities. Similarly, Brescia et al. [25] enrolled consecutive patients, reflecting specific clinical practices that may limit generalizability. Such selection methods can skew the results and affect the applicability of the findings to the general population.

Inconsistencies in measuring clinical outcomes introduce measurement bias. For example, Okushi et al. [14] used different diagnostic techniques to assess polyp recurrence, and DeConde et al. [18] relied on subjective symptom reporting. These variations can lead to differential accuracy and reliability of the data collected. Van Zele et al. [15] employed a mix of objective and subjective measures, which might not be uniformly applied, further contributing to measurement bias. The lack of standardized measurement protocols across studies complicates the comparison of results and emphasizes the need for uniform diagnostic criteria.

Confounding variables were not adequately controlled in several studies. Meng et al. [20] did not account for differences in postoperative care, and Vlaminck et al. [16] did not adjust for variations in patient adherence to medical therapy. Brescia et al. [25] noted associations with age and sex but did not consider other potential confounders like environmental factors or genetic predispositions. The failure to control for these variables can obscure the true relationship between predictors and recurrence, leading to biased estimates.

Studies such as those by Rosati et al. [22] and Bai et al. [24] that rely on patient self-reports for symptom recurrence are susceptible to recall bias. Patients may underreport or overreport symptoms and medical events, affecting the study's accuracy. This bias can be particularly problematic in long-term follow-up studies, where patients' memories of past symptoms and treatments may not be reliable. Objective data collection methods are needed to mitigate recall bias and improve the reliability of the findings.

Longitudinal studies, including those by Mohnsenh et al. [19] and Salvador et al. [27], face the risk of attrition bias due to extended follow-up periods. Patients lost to follow-up might have different recurrence rates or characteristics than those who remain in the study, potentially skewing the results. Ensuring complete follow-up data and accounting for dropouts in the analysis are essential to addressing this bias.

Subjective assessments of polyp recurrence and severity can introduce observer bias, as seen in the studies by Beswick et al. [23] and Sella et al. [21]. Differences in clinical judgment and diagnostic criteria can lead to variability in outcome reporting. Salvador et al. [27] used specific diagnostic criteria, which may differ from those used by other researchers, further complicating cross-study comparisons. Training and calibration of clinicians involved in these studies can help reduce observer bias.

To address these biases, future research should implement several strategies. Transparent and inclusive selection criteria can ensure a representative patient population, reducing selection bias. Utilizing uniform diagnostic criteria and measurement methods across studies can minimize measurement bias and enhance comparability. Conducting multivariate analyses to account for potential confounding variables can provide a clearer understanding of the predictors of recurrence. Employing validated questionnaires and electronic health records can mitigate recall and reporting bias. Implementing strategies to minimize loss to follow-up and ensure complete data collection is essential. Standardizing diagnostic and assessment procedures through training can reduce observer bias and improve the consistency of clinical assessments.

Strengths and Limitations

This comprehensive systematic review describes in detail the risk factors for the recurrence of CRSwNP. The fact that there was significant heterogeneity among the research endpoints that were included may be a limitation. This can be a result of the observational data being used. Although randomization is not an issue with observational studies, the selection of controls may lead to bias, as discussed earlier. Another limitation refers to the quality of the included studies, which also varied. For more conclusive evidence, more studies with similar endpoints are needed. Not all of the included studies contained information on aspects including the time to relapse as well as related issues such as population T-helper cell profile, follow-up duration, and environmental factors. For instance, not all risk factors, such as participant gender, congruent use of medication, and smoking, that might have affected the disease course were consistently reported.

## Conclusions

Chronic rhinosinusitis with nasal polyposis can impose a significant burden on patients, healthcare systems, and society and has been found to be highly recurrent post ESS. Asthma, aspirin intolerance, peripheral eosinophilia, IL-5 expression, T2 profile, and intense sinus opacification have been noted as independent predictors of the condition in different studies. Age and gender have also been found to interact, resulting in its onset and progression. This is despite congruent medical management in many cases. Therefore, recurrent polyposis in CRS signals a more aggressive disease course, requiring close follow-up and revision surgeries. Effective management of CRSwNP can reduce the burden of the disease by improving symptom control, preventing exacerbations, using image-guided navigation systems, and reducing healthcare resource utilization.
